# Doxorubicin concentrations in bone tumour-relevant tissues after bolus and continuous infusion: a randomized porcine microdialysis study

**DOI:** 10.1007/s00280-023-04637-1

**Published:** 2024-02-08

**Authors:** Andrea René Jørgensen, Mats Bue, Pelle Hanberg, Elisabeth Krogsgaard Petersen, Christina Harlev, Jakob Hansen, Thomas Baad-Hansen, Akmal Safwat, Maiken Stilling

**Affiliations:** 1 Aarhus Microdialysis Research Group, Orthopaedic Research Unit, Aarhus University Hospital, Palle Juul-Jensens Boulevard 99, J112, 8200 Aarhus N, Denmark; 2 Department of Clinical Medicine, Aarhus University, Aarhus N, Denmark; 3 Department of Orthopaedic Surgery, Aarhus University Hospital, Aarhus N, Denmark; 4 Department of Forensic Medicine, Aarhus University, Aarhus N, Denmark; 5 Department of Oncology, Aarhus University Hospital, Aarhus N, Denmark

**Keywords:** Microdialysis, Doxorubicin, Doxorubicinol, Infusion, Bone Cancer

## Abstract

**Purpose:**

Doxorubicin is a widely used chemotherapeutic drug that can be administered intravenously as both a bolus infusion and a continuous infusion. The latter is believed to lower the risk of cardiotoxicity, which is a critical long-term complication of doxorubicin treatment. The local tissue concentrations of doxorubicin will be reflected in both treatment efficacy and toxicity, but very limited information is available. The aim of this study was to measure the concentration of doxorubicin after continuous and bolus infusion in tissue compartments around a typical location of a bone tumour.

**Methods:**

Sixteen pigs (female, Danish Landrace, mean weight 77 kg) were randomized into two groups of eight. Both groups received an intravenous infusion of 150 mg doxorubicin; Group 1 received a bolus infusion (10–15 min) and Group 2 received a continuous infusion (6 h). Before infusion, microdialysis catheters were placed intravenously and in four bone tumour-relevant tissue compartments (cancellous bone, subcutaneous tissue, synovial fluid of the knee joint and muscle tissue). Sampling was done (*n* = 15) over 24 h, and venous blood samples were collected as a reference.

**Results:**

Area under the concentration–time curve (AUC_0–24 h_) for plasma (total concentration) was significantly different between the two groups, while peak drug concentration (*C*_max_) was significantly higher in two compartments (plasma and synovial fluid of the knee joint) in Group 1 compared to Group 2. Overall, the unbound tissue concentrations were extremely low with values below 0.20 µg/mL.

**Conclusion:**

The pharmacokinetic profile for doxorubicin in the investigated tissues is very similar when comparing bolus and 6 h continuous infusion.

**Supplementary Information:**

The online version contains supplementary material available at 10.1007/s00280-023-04637-1.

## Introduction

Doxorubicin is a frequently used chemotherapeutic agent originating from the anthracycline family. Despite its clinical use for more than 50 years, the cytotoxic actions are still being debated, but are often ascribed to the inhibition of the topoisomerase enzyme II as well as the formation of reactive oxygen species [1–5]. Doxorubicin is used in both treatment and palliative therapy for a broad spectrum of cancers, e.g., osteosarcoma, breast cancer and leukaemia [1]. The use of doxorubicin can be both monotherapeutic and in combination with other chemotherapeutic drugs, radiation and/or surgery. When used as monotherapy or in combination with surgery, the recommended dosage is 60–75 mg/m^2^, based on the patient body surface area, and reduced in the case of compromised kidney and/or liver function [1]. Furthermore, it can be adjusted according to the level of effect as well as the development of side effects.

The introduction of doxorubicin-based combination chemotherapy has, together with improved surgical interventions, contributed significantly to the survival of cancer patients, e.g., the 5-year survival for patients with osteosarcoma has since the 1960s increased from 30 to 80% [6]. Despite the long history of doxorubicin usage, only limited information regarding local target tissue concentrations of the drug exists. Until now, the current knowledge regarding tissue concentrations is merely based on analysis of tissue specimens, whose value is limited because of poor time resolution and not being able to distinguish between the bound and unbound fraction. Microdialysis is a promising pharmacokinetic tool that allows for continuous and simultaneous sampling of unbound molecules and can therefore circumvent these challenges.

Intravenously administered doxorubicin can be given as either bolus or continuous infusion. Continuous infusion is believed to reduce the risk of cardiotoxicity, which is a devastating and dose-limiting side effect [7, 8]. Studies measuring the concentration of doxorubicin in plasma following systemic bolus administration have found great inter- and intraindividual differences [9–11]. These findings in combination with the potential risk of side effects, whereof the metabolite doxorubicinol is believed to contribute to the risk of cardiotoxicity [12–14], underline the need for also investigating local target tissue concentrations of doxorubicin and doxorubicinol to possibly correlate target concentrations with both effect and toxicity.

The aim of this porcine study was to apply microdialysis for the assessment of doxorubicin concentrations in bone tumour-relevant tissues: cancellous bone, subcutaneous tissue, synovial fluid of the knee joint, muscle tissue and in the blood after bolus and continuous intravenous administration. We hypothesized heterogeneous distribution between the investigated tissue compartments, with lower concentrations in the bone tissue compared to soft tissue and higher peak drug concentrations in the bolus group compared to the continuous infusion group.

## Materials and methods

### Ethical approval

The study was conducted at the Institute of Clinical Medicine, Aarhus University, Aarhus, Denmark. Approval was obtained from the Danish Animal Experiments Inspectorate (license no. 2017/15–0201-01184) and carried out in accordance with existing laws and ARRIVE guidelines. All chemical analyses were performed at the Department of Forensic Medicine, Aarhus University, Aarhus, Denmark.

### Microdialysis

Microdialysis is a catheter-based method enabling dynamic collection of small samples, from any tissue of interest, called dialysates. This allows for concentration quantification of virtually any unbound drug in the extracellular matrix with a size below the membrane cut-off. The basic principle behind microdialysis is passive concentration-driven diffusion, which happens across a semipermeable membrane located at the tip of the microdialysis catheter. An equilibrium between the membrane and the surrounding tissue will never occur as the catheter is connected to a precision pump that continuously perfuses the system with a perfusion fluid at a set flow rate. This means that the concentration measured in the dialysate only represents a fraction of the absolute concentration in the tissue of interest. The fraction is referred to as the relative recovery and can be determined by several calibration methods [15]. In the present study, calibration by drug was applied meaning that after the observation period, a known concentration of a doxorubicin solution replaced the initial perfusion fluid, and two 40 min dialysates were collected under this setup.

The relative recovery (RR) could then be determined by the following equation [16, 17]:

$${\text{RR}}= \frac{{C}_{{\text{perfusate}}}- {C}_{{\text{dialysate}}}}{{C}_{{\text{perfusate}}}}$$

*C*_perfusate_ is the concentration of doxorubicin in the perfusate during the calibration period, while *C*_dialysate_ is the concentration of doxorubicin in the dialysate during the calibration period.

The relative recovery was then used to calculate the absolute tissue concentration of unbound doxorubicin (*C*_tissue_):

$${C}_{{\text{tissue}}}=\frac{{C}_{{\text{dialysate}}}}{{\text{RR}}}$$

*C*_dialysate_ is the concentration of doxorubicin in the dialysate during the sampling period.

In a standard microdialysis setup, dialysates are collected in 200 μl microvials. However, in the present study, the collection was done in 1.5 ml LoBind Eppendorf tubes (Eppendorf, Hamburg, Germany) as thorough prior in vitro and in vivo experiments have shown that undesirable adsorption of doxorubicin was caused almost solely by the standard polystyrene/santoprene vials [18].

The microdialysis equipment was purchased from M Dialysis AB (Stockholm, Sweden). All microdialysis catheters had a cut-off of 20 kDa. The catheters were type 70 with membrane lengths of 20 mm and 30 mm and type 67 intravenous catheters with 30 mm membranes. The flow rate was 1 μl/min, and the perfusion fluid was saline. The concentration of the doxorubicin solution used under the calibration period was determined as the mean of three samples from the solution.

### Animals and anaesthesia

Sixteen female pigs (Danish Landrace, mean weight 77 kg (range 73–83 kg), age 5 months) were included in the study and randomized into two groups of eight. The animals were kept in pens in groups of minimum two animals with a light cycle of 12 h. Straw was used as bedding, and they had access to ad libitum water. Feeding was restricted (farm pig ration) to limit weight gain. Before transportation to the surgical facility, the animals were sedated with zoletil mix ((25 mg/ml tiletamine + 25 mg/ml zolazepam) + 6.25 ml xylazine (20 mg/ml) + 1.25 ml ketamine (100 mg/ml) + 2.5 ml butorphanol (10 mg/ml) 1 ml/10 kg)). Upon arrival, the animals were placed under general anaesthesia and kept so until euthanasia with an overdose of pentobarbital at the end of the sampling period. The anaesthesia consisted of a combination of continuous intravenous infusion of propofol (40 ml/h) (Fresenius Kabi, Bad Homburg, Germany) and fentanyl (25 ml/h) (B. Braun, Melsungen, Germany). Arterial blood gas samples were taken and analysed every 2 h to monitor pH, which was within a range of pH 7.38–7.57. Body temperature was controlled with a rectal thermometer and regulated by room temperature, ventilation, cooling fluids and covers.

To minimize the risk of the anaesthesia and long observation period to affect the distribution of doxorubicin, a minimal accepted mean arterial pressure (MAP) of 65 mmHg was opted for. For animals going below this value, continuous infusion of norepinephrine (concentration: 0.1 mg/mL, start infusion rate: 0.3 mL/h) was started. The infusion rate was increased by 0.3 mL/h according to need. The animals were continuously provided with fluid to control both the level of glucose and urine production. The blood loss during the surgery was minimal.

### Randomization

Before any surgical intervention, the animals were block-randomized in pairs of two to receive either bolus or continuous administration of doxorubicin. Randomization was done by drawing a note indicating *Group 1* (bolus administration) or *Group 2* (continuous administration) from a non-translucent envelope.

### Surgical procedures

After induction of anaesthesia, surgical procedures were initiated. With the pig in a supine position, a central venous catheter was placed ultrasound-guided in a jugular vein. Via an approximately 5–6 cm midline incision starting from 2–3 cm cranial to manubrium sterni, an arterial sheath was placed in the internal carotid artery on the opposite side.

**Fig. 1:**
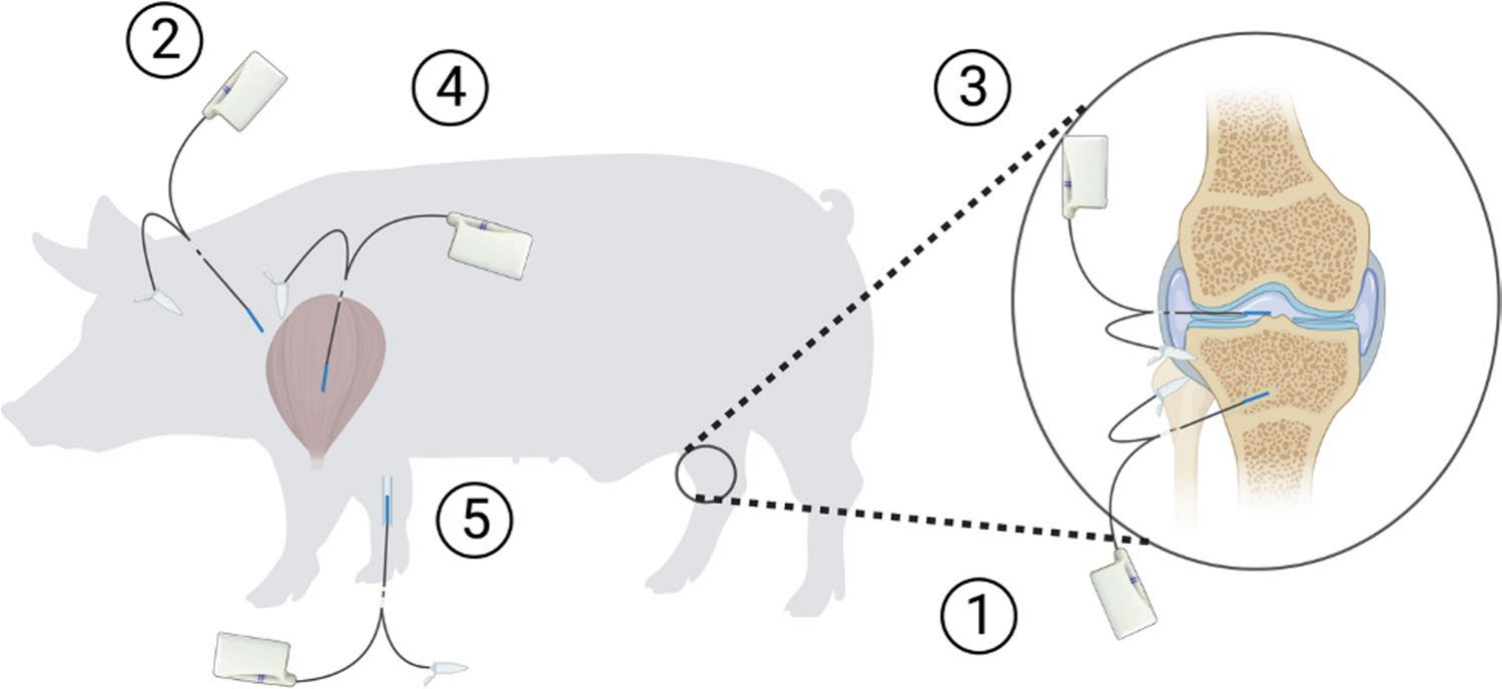
Location of microdialysis catheters; (1) cancellous bone, (2) subcutaneous tissue, (3) synovial fluid of the knee joint, (4) muscle tissue and (5) intravenously

With an anteromedial incision starting approximately 2 cm below the tibial plateau and continuing to the midpoint of the anterior crest, the right tibial bone was assessed. A drill hole, 35 mm in depth and ∅2 mm, was made in the cancellous bone approximately 10 mm distal to the epiphyseal line. Overheating of the bone was prevented by frequent pausing and continuous cooling with saline. A microdialysis catheter, with a membrane length of 30 mm, was placed in the cancellous drill hole (Fig. 1).

A 30-mm catheter was placed in the synovial fluid of the right knee joint using a splittable introducer. Also, by the use of splittable introducers and ultrasound, a 30-mm catheter was placed in the muscle tissue (mean depth 104 mm, range 94–116 mm) and subcutaneous tissue on the right front leg, respectively. Finally, a 30 mm intravenous catheter (type 67) was placed on the right front leg to assess unbound plasma concentrations. All microdialysis catheters were sutured to the skin for fixation. The positions of the synovial fluid of the knee joint and cancellous bone catheters were verified intraoperatively with the use of fluoroscopic imaging. After euthanasia, the drill holes in the cancellous bone were verified by computed tomography (CT).

After the placement of all catheters, each catheter was connected to a precision pump. To fill the entire microdialysis system with perfusion fluid (saline), flushing was performed until no air bubbles seemed to be trapped within the system.

### Administration of doxorubicin

To lower the risk of extravasation, 500 mL of saline was administered through the central venous catheter over 30 min before the administration of doxorubicin, as in accordance with clinical guidelines. Hereafter, Group 1 received an intravenous bolus administration of 150 mg doxorubicin over 10–15 min, while Group 2 received a continuous administration of 150 mg doxorubicin over 6 h. Both administrations were followed by an administration of a minimum of 100 mL saline.

Every animal received a dosage of 150 mg of doxorubicin, and dosage was thus not determined by body surface area due to a lack of existing formulas for the specific porcine breed [19, 20].

### Sampling

**Fig. 2:**
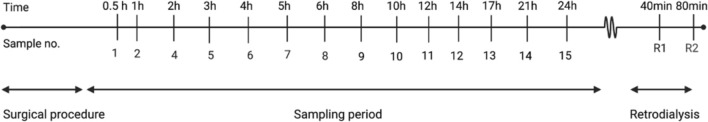
Overview of sampling

The initiation of administration of doxorubicin is defined as time zero (*T* = 0) (Fig. 2). The overall sampling period was 24 h. In both groups, dialysates were collected every 30 min from time 0 to 120 min, every 60 min from time 120 min to 360 min and every 120 min from time 360 min to 840 min. At time 960 min, 1200 min and 1380 min, a LoBind Eppendorf tube was placed for collection of dialysates over 60 min. A total of 15 dialysates were collected from each compartment in each animal. After the collection of the last dialysate, calibration was performed with the collection of two calibration dialysates. Calibration was performed with a solution containing 10 μg/mL doxorubicin hydrochloride. Blood samples were drawn from a central venous catheter at the midpoint of each dialysate sampling interval. A total of 15 blood samples were taken. During the entire sampling period, the lights in the operation room were switched off due to the risk of photodegradation of doxorubicin.

### Handling of samples

All dialysates were stored immediately after collection at -80 °C until analysis. Venous blood samples (EDTA 1.8 mg/mL) were stored for a maximum of 2 h before being centrifuged at 3000*g*, for 10 min at 5 °C. After centrifugation, plasma samples were stored at -80°°C until analysis.

### Quantification of doxorubicin in microdialysates and plasma samples by ultra-high performance liquid chromatography and tandem mass spectrometry (UHPLC-MS/MS)

Doxorubicin and doxorubicinol were quantified in microdialysate and blood plasma samples by UHPLC-MS/MS using a previously described and validated method [18]. The method utilizes stable isotope-labelled doxorubicin (13CD3-doxorubicin) as internal standard and a linear calibration model based on matrix-correct calibrator samples spiked with reference standard compounds (see Supplemental Fig. 1 for selected method documentation and further details in [18]).

The lower limit of quantification for doxorubicin and doxorubicinol was estimated to be 0.002 (dialysate) and 0.003 µg/mL (plasma), respectively, and standard requirements for the method precision (CV < 15%) and trueness (bias < 15%) were met.

#### Pharmacokinetic analysis and statistics

All concentrations quantified in the dialysate are of unbound doxorubicin, while the plasma samples represent the total concentrations (bound + unbound). For all animals and each compartment, the following pharmacokinetic parameters were calculated by non-compartmental analysis using Stata (version 16.0, StataCorp, College Station, Texas, USA): area under the concentration–time curve (AUC_0–24 h_) from time zero until 24 h, peak drug concentration (*C*_max_), time to peak drug concentration (*T*_max_) and tissue penetration AUC_tissue_/AUC_plasma_. The AUC_0–24 h_ was calculated by the use of the linear up-log-down trapezoidal method. The *C*_max_ was calculated as the mean peak concentration of doxorubicin in each compartment. The *T*_max_ was estimated as time until *C*_max_. All measured dialysate doxorubicin concentrations were attributed to the midpoint of each sampling interval. The pharmacokinetic parameters for doxorubicin between the two groups were compared using mixed models for repeated measurements taking into account multiple compartments per animal, followed by post-hoc tests for pairwise comparisons. All model assumptions were tested by visual inspection of residuals, fitted values and estimates of random effects. Due to a lack of normal distribution, log-transformed T_max_ for doxorubicin was analysed, and the results were back-transformed giving medians and ratio of medians for comparisons.

The pharmacokinetic parameters for doxorubicinol between the two groups were compared by *t*-test.

A *p*-value < 0.05 was regarded as statistically significant.

#### Target evaluation

IC50 is the concentration capable of inhibiting 50% of a tumour cell line. Time above IC50 for two selected osteosarcoma cell lines (HOS: 0.016536531 μg/mL and NOS-1: 0.046344245 μg/mL) was calculated by linear interpolation in Microsoft Excel [21].

## Results

### Relative recoveries

The ranges of mean relative recovery (SD) for Group 1 and Group 2 were 44% (17)—84% (7) and 26% (24)—72% (7), respectively.

### Animal completion

With the exception of one animal from each group, all animals survived the entire study period. The animal from Group 1 died in relation to surgery before any samples could be taken. The animal in Group 2 died between samples 13 and 14. For this animal, a mean relative recovery based on the relative recovery from the animals from the same group was applied for each compartment.

### Pharmacokinetic parameters

Pharmacokinetic parameters are presented in Table 1. The AUC_0–24 h_ was similar between the two groups, except for plasma (total concentration), which was higher in Group 2. *C*_max_ was higher for plasma and synovial fluid of the knee joint in Group 1. *T*_max_ showed a high level of variance, but there was a tendency towards faster *T*_max_ in Group 1, but this was not statistically significant for two compartments; cancellous bone and synovial fluid of the knee joint, the latter being almost the same between the two groups. Except for synovial fluid of the knee joint, there was no difference in tissue penetration between the two groups.

**Table 1:** Pharmacokinetic parameters for doxorubicin

Pharmacokinetic parameters	Group 1 (bolus)		Group 2 (continuous)		Comparison	
					Difference (95% CI)	*P*-value
**AUC**_**0–24 h**_**, min μg/mL mean (95%CI)**						
Plasma	*N* = 7	36.8 (19.5; 54.0)	*N* = 8	69.1 (53.0; 85.2)	-32.3 (-56.0; -8.7)	0.007*
Intravenous	*N* = 7	14.9 (6.9; 23.0)	*N* = 8	12.5 (4.9; 20.0)	2.5 (-8.6; 13.5)	0.641
Subcutaneous tissue	*N* = 7	5.7 (1.1; 10.2)	*N* = 8	6.9 (2.7; 11.2)	-1.3 (-7.5; 5.0)	0.692
Muscle	*N* = 7	3.4 (1.0; 5.8)	*N* = 8	4.2 (2.0; 6.5)	-0.8 (-4.1; 2.5)	0.603
Synovial fluid of the knee joint	*N* = 7	7.7 (5.0; 10.4)	*N* = 8	6.8 (4.3; 9.3)	0.9 (-2.7; 4.6)	0.606
Cancellous bone	*N* = 7	1.0 (-1.4; 3.4)	*N* = 8	2.6 (0.35; 4.9)	-1.6 (-4.9; 1.7)	0.320
***C***_**max**_**, μg/mL mean (95%CI)**						
Plasma	*N* = 7	0.86 (0.69; 1.03)	*N* = 8	0.24 (0.08; 0.40)	0.6 (0.4; 0.8)	0.000*
Intravenous	*N* = 7	0.08 (0.04; 0.13)	*N* = 8	0.03 (-0.01; 0.07)	0.06 (-0.004; 0.1)	0.064
Subcutaneous tissue	*N* = 7	0.03 (0.02; 0.05)	*N* = 8	0.02 (0.002; 0.03)	0.02 (-0.003; 0.04)	0.090
Muscle	*N* = 7	0.01 (0.003; 0.02)	*N* = 8	0.01 (0.01; 0.02)	-0.003 (-0.01; 0.01)	0.484
Synovial fluid of the knee joint	*N* = 7	0.09 (0.04; 0.15)	*N* = 8	0.02 (-0.03; 0.06)	0.08 (0.01; 0.2)	0.034*
Cancellous bone	*N* = 7	0.004 (-0.004; 0.01)	*N* = 8	0.01 (0.003; 0.02)	-0.01 (-0.02; 0.01)	0.289
***T***_**max**_**, min median (min; max)**						
Plasma	*N* = 7	15.0 (15; 15)	*N* = 8	203 (15; 540)	0.07 (0.02; 0.26)	0.000*
Intravenous	*N* = 7	34 (15; 105)	*N* = 8	139 (15; 420)	0.24 (0.74; 0.80)	0.023*
Subcutaneous tissue	*N* = 7	30 (15; 75)	*N* = 8	125 (15; 330)	0.24 (0.06; 0.96)	0.044*
Muscle	*N* = 7	51 (45; 105)	*N* = 8	93 (15; 330)	0.55 (0.19; 1.58)	0.246*
Synovial fluid of the knee joint	*N* = 7	21(15; 45)	*N* = 7	62 (15; 330)	0.33 (0.10; 1.12)	0.073
Cancellous bone	*N* = 4	60 (15; 210)	*N* = 5	62(15; 420)	0.93 (0.25; 3.43)	0.914
**AUC**_**tissue**_**/AUC**_**plasma**_** mean (95% CI)**						
Intravenous	*N* = 7	0.37 (0.20; 0.54)	*N* = 8	0.20 (0.04; 0.36)	0.17 (-0.07; 0.4)	0.149
Subcutaneous tissue	*N* = 7	0.15 (0.06; 0.24)	*N* = 8	0.12 (0.03; 0.20)	0.04 (-0.08; 0.15)	0.562
Muscle	*N* = 7	0.09 (0.04; 0.13)	*N* = 8	0.06 (0.02; 0.11)	0.02 (-0.04; 0.09)	0.445
Synovial fluid of the knee joint	*N* = 7	0.22 (0.16; 0.28)	*N* = 8	0.10 (0.05; 0.16)	0.12 (0.04; 0.20)	0.005*
Cancellous bone	*N* = 7	0.03 (-0.01; 0.07)	*N* = 8	0.04 (0.01; 0.08)	-0.01 (-0.07; 0.04)	0.610

*Indicates a statistically significant difference, *p*-value < 0.05

A comparison of the compartments within each group showed that there was a statistically significant difference between AUC_0–24 h_, *C*_max_ and *T*_max_ between many compartments (Table 2).

**Table 2:** Comparison of pharmacokinetic parameters within groups

	Group 1 (bolus) (*p*-value)			Group 2 (continuous) (*p*-value)		
	AUC_0–24 h_	C_max_	T_max_	AUC_0–24 h_	C_max_	T_max_
Plasma vs. subcutaneous tissue	0.001*	0.000*	0.146	0.000*	0.005*	0.283
Plasma vs. cancellous bone	0.000*	0.000*	0.002*	0.000*	0.004*	0.006*
Intravenous vs. plasma	0.022*	0.000*	0.039*	0.000*	0.009*	0.311
Intravenous vs. subcutaneous tissue	0.039*	0.033*	0.785	0.176	0.580	0.790
Intravenous vs. muscle	0.008*	0.003*	0.163	0.033*	0.436	0.127
Intravenous vs. synovial fluid of the knee joint	0.076	0.769	0.158	0.129	0.705	0.022*
Intravenous vs. cancellous bone	0.002*	0.002*	0.185	0.014*	0.413	0.041*
Muscle vs. plasma	0.000*	0.000*	0.000*	0.000*	0.004	0.012*
Muscle vs. subcutaneous tissue	0.305	0.001*	0.185	0.195	0.564	0.414
Muscle vs. cancellous bone	0.017*	0.205	0.696	0.087	0.772	0.239
Synovial fluid of the knee joint vs. plasma	0.001*	0.000*	0.435	0.000*	0.007*	0.002*
Synovial fluid of the knee joint vs. subcutaneous tissue	0.381	0.031*	0.393	0.942	0.982	0.106
Synovial fluid of the knee joint vs. muscle	0.000*	0.002*	0.002*	0.021*	0.895	0.161
Synovial fluid of the knee joint vs. cancellous bone	0.000*	0.001*	0.011*	0.000*	0.864	0.990
Cancellous bone vs. subcutaneous tissue	0.036*	0.000*	0.181	0.039*	0.512	0.138*

*Indicates a statistically significant difference, *p*-value < 0.05

### Concentrations

**Fig. 3:**
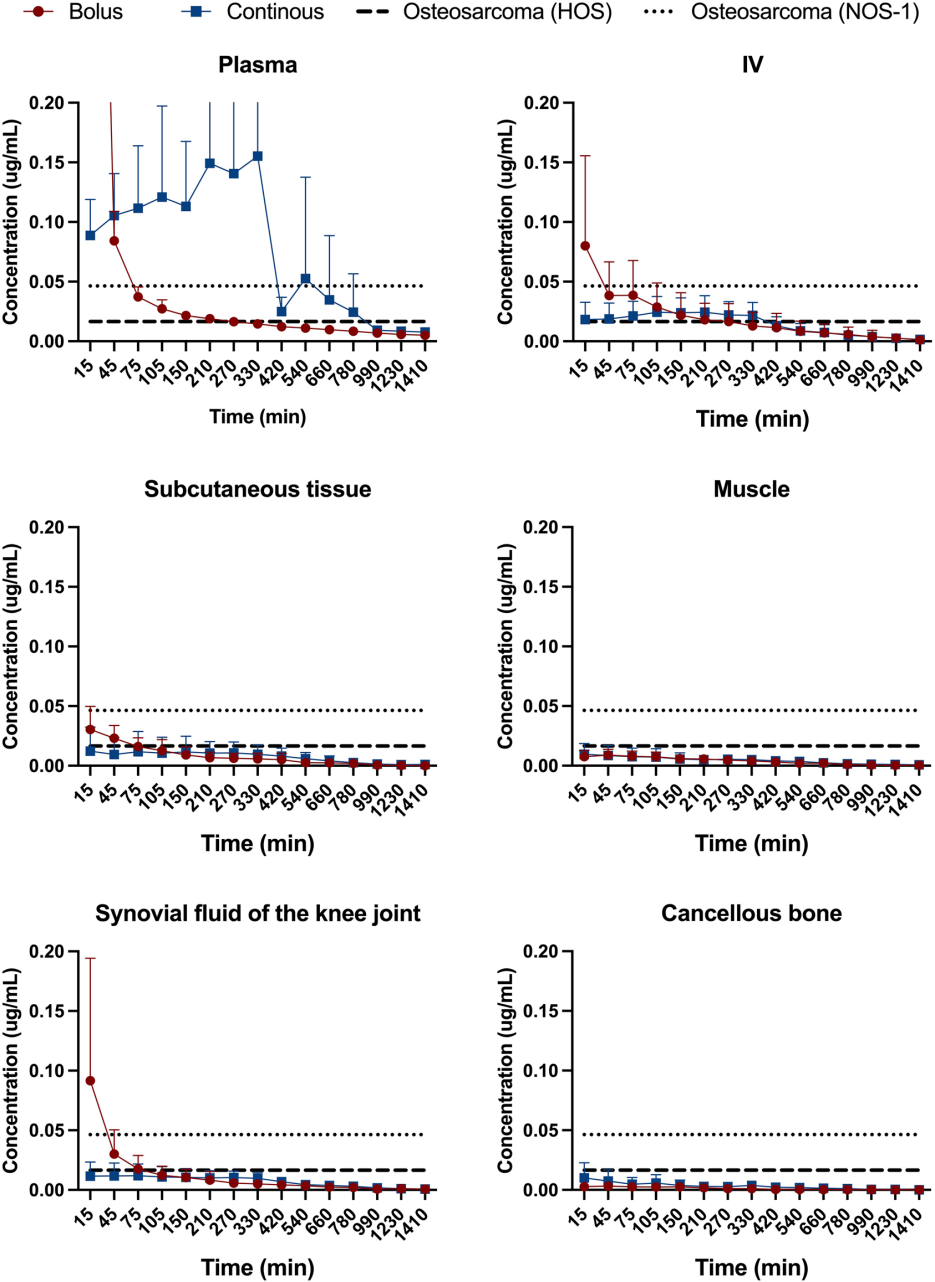
Concentration–time profile of doxorubicin after bolus (red) and continuous (blue) administration. The dotted lines indicate IC50 for osteosarcoma HOS (0.0165 μg/ml) and osteosarcoma NOS-1 (0.0463 μg/ml). Plasma represents the total concentration (both bound and free doxorubicin), while IV (intravenous) only represents the unbound fraction

Figure 3 depicts six concentration–time graphs comparing the two groups for each compartment. Overall, the unbound tissue concentrations were extremely low with values below 0.20 µg/mL. In each graph, two horizontal lines indicating the IC50 for the two cell lines, osteosarcoma HOS and NOS-1, are inserted. Time above IC50 (*T* > IC50) for the two cell lines osteosarcoma HOS and NOS-1 is presented in Table 3. A varying *T* > IC50 was observed both within and between the compartments illustrated by wide min and max range. Overall, the concentrations measured were very low.

**Table 3:** *T* > IC50 for two osteosarcoma cell lines

	Group 1 (bolus)		Group 2 (continuous)		*p*-value
**T > IC50 (min) (min; max) for osteosarcoma HOS, 0.016536531 μg/mL**					
Intravenous	*N* = 7	283 (0; 965)	*N* = 8	293 (0; 687)	0.917
Subcutaneous tissue	*N* = 7	72 (0; 196)	*N* = 8	79 (0; 629)	0.027*
Muscle	*N* = 7	0 (0; 0)	*N* = 8	33 (0; 116)	0.736
Synovial fluid of the knee joint	*N* = 7	79 (0; 241)	*N* = 8	69 (0; 272)	0.921
Cancellous bone	*N* = 7	0 (0; 0)	*N* = 8	16 (0; 88)	0.873
Plasma	*N* = 7	271 (174; 415)	*N* = 8	581 (415; 968)	0.002*
**T > IC50 (min) (min; max) for osteosarcoma NOS-1, 0.046344245 μg/mL**					
Intravenous	*N* = 7	57 (0; 235)	*N* = 8	10 (0; 41)	0.260
Subcutaneous tissue	*N* = 7	3 (0; 24)	*N* = 8	20 (0; 157)	0.696
Muscle	*N* = 7	0 (0; 0)	*N* = 8	0 (0; 0)	1.000
Synovial fluid of the knee joint	*N* = 7	20 (0; 60)	*N* = 8	0 (0; 0)	0.633
Cancellous bone	*N* = 7	0 (0; 0)	*N* = 8	0 (0; 0)	1.000
Plasma	*N* = 7	69 (53; 99)	*N* = 8	454 (374; 864)	0.000*

*Indicates a significant difference, *p*-value < 0.05

### Doxorubicinol

Table 4 shows the pharmacokinetic parameters for the metabolite doxorubicinol, which was only detectable in plasma in both groups. Both AUC_0-24 h_ and C_max_ were significantly higher in Group 1 compared to Group 2.

**Table 4:** Pharmacokinetic parameters for the metabolite doxorubicinol in plasma

	Group 1 (bolus)		Group 2 (continuous)		Difference (95% CI)	*p*-value
AUC_0-24 h_ min μg/mL, mean (95%CI)	*N* = 7	3.0 (2.1; 4.0)	*N* = 8	0.8 (0.3; 1.3)	2.3 (1.3; 3.2)	0.0005*
C_max_, μg/mL, mean (95%CI)	*N* = 7	0.007 (0.006; 0.009)	*N* = 8	0.003 (0.002; 0.007)	0.004 (0.003; 0.006)	0.001*

*Indicates a statistically significant difference, *p*-value > 0.05

## Discussion

The aim of the present study was to evaluate doxorubicin concentrations in bone tumor-relevant tissues, comparing the concentrations after a bolus and 6 h continuous infusion during a 24 h sampling period. Our key findings were that bone and muscle compartments had a similar pharmacokinetic profile of doxorubicin for the two administration forms. For plasma and synovial fluid of the knee joint, the mean *C*_max_ was significantly higher for the bolus group, while mean plasma AUC_0–24 h_ was higher after continuous infusion. In contrast to the hypothesis, the distribution of doxorubicin in the different compartments was fairly homogenous, and peak drug concentrations were only higher in the bolus group for selected compartments.

### Pharmacokinetic target

It is complicated to correlate doxorubicin target tissue concentrations with clinical effects due to a lack of knowledge regarding efficient pharmacokinetic/pharmacodynamic (PK/PD) targets. One potential target is the IC50, which indicates the concentration capable of inhibiting 50% of a tumour cell line. However, the IC50 target is an in vitro defined target and may therefore not be directly translational to in vivo settings. In addition, multiple cancer cell lines exist for each cancer type, and they all have varying IC50. In the clinic, it is not standard to determine the specific tumour cell line, e.g. all osteosarcomas are treated identically. Furthermore, there is no defined goal for time, AUC_0–24 h_ or *C*_max_ above IC50.

One approach could be to aim for the highest IC50, which for osteosarcoma would be the G-292-clone-A141B1 cell line with an identified IC50 of 1.43 μg/mL [22]. This is more than 80 times higher than the lowest IC50 value presented in Fig. 3 (0.017 μg/mL) and higher than the measured *C*_max_ in both groups (Group 1 mean range: 0.01–0.83 μg/mL; Group 2 mean range: 0.01–0.18 μg/mL). Aiming for the highest IC50 could also lead to overtreatment in individuals with tumour cell lines sensitive to lower concentrations and therefore also increase their risk of toxicities. However, in vitro studies on different cell lines (neoplastic and regular) have shown a decreased cell survival about both increased doxorubicin concentrations and exposure time [23, 24], and even a decrease in IC50 has been described with increasing exposure time [24]. Based hereupon, the results presented should be interpreted with caution in relation to the IC50 target. Future studies investigating the correlation between local tissue concentrations and clinical effects are warranted.

### Cardiotoxicity

When comparing bolus and continuous administration, other studies, both experimental and clinical, have also found a statistically significantly higher *C*_max_ in plasma (total) following bolus administration and a similar AUC [25, 26]. The course of cardiotoxicity is still debated but is often ascribed to higher plasma *C*_max_, of both doxorubicin and doxorubicinol, which is supported by the fact that several clinical studies have found a lower risk of cardiotoxicity after continuous infusion compared to bolus infusion in adults [7, 25]. In the present animal study, a 6 h continuous infusion was chosen, as infusion duration of 6 h or longer has been shown to significantly lower the risk of clinical heart failure following doxorubicin treatment [8]. However, some studies have found no cardioprotective benefits based on a 48 h continuous doxorubicin infusion compared to bolus infusion in childhood high-risk acute lymphoblastic leukaemia (ALL) patients [27, 28]. These studies concluded that continuous infusion should not be applied for the sole reason of reducing the risk of cardiotoxicity for the investigated study population [27]. The reason is a supposed higher risk of drug extravasation leading to tissue necrosis and infection in addition to possible overlapping periods of myelosuppression when applying continuous infusion. [7].

### Alternative applications

The ideal treatment with doxorubicin is the attainment of sufficient concentrations in tissues affected by cancer and very low to no doxorubicin in the remaining healthy tissue. Therefore, alternative administration forms, such as liposomal doxorubicin (encapsulated doxorubicin administered intravenously), and local applications, such as isolated limb perfusion, are being investigated [29–35]. Microdialysis could serve as a suitable tool to evaluate the doxorubicin pharmacokinetics course of these application methods as well as the potential of local applications.

### Limitations

Bone tumour-relevant tissues were chosen as target tissues in the current study to facilitate the clinical translation potential of the results to patients with bone sarcoma. However, none of the animals had any tumours. Previous studies have shown a heterogeneous distribution of chemotherapeutic agents within solid tumours, wherefore the concentration to all kinds of tissues is still of utmost interest, not least to evaluate the possible level of toxicity [36, 37]. Pigs are considered a good experimental model due to many resemblances with human physiology, such as enzymatic activity and anatomy [19, 38]. However, due to the young age of the animals used in the present study, the maturation state of the bone (persistent epiphyseal line) may best approximate that of a human child. Penetration may therefore be different compared to the human adult bone. Another factor possibly affecting penetration was the long anaesthesia time. However, all animals were kept above an MAP of 65 mmHg throughout the entire study period.

A direct comparison to plasma values found in clinical studies is complicated by different administration times as well as dosing. An additional limitation related to microdialysis is the risk of magnification of data variation associated with the pre-analytical handling and sampling assay when correcting for relative recovery. The variation increases with decreasing relative recovery. However, all mean relative recoveries in the present study were well above 20%, which is considered the critical value [16].

## Conclusion

With the use of the microdialysis technique, similar pharmacokinetic profiles of doxorubicin were found in the investigated bone tumour-relevant tissues after bolus and 6 h continuous infusion. Mean *C*_max_ was higher in two compartments (plasma and synovial fluid of the knee joint) in the bolus group, while mean plasma AUC_0–24 h_ was higher after continuous infusion. AUC_0–24 h_ and *C*_max_ for the metabolite doxorubicinol were significantly higher in the bolus group.

### Supplementary Information

Below is the link to the electronic supplementary material.

Supplementary file1 (PDF 542 KB)

## Data Availability

The data of this study are available from the corresponding author upon reasonable request.
