# Supplementary material for: Doxorubicin concentrations in bone tumour-relevant tissues after bolus and continuous infusion: a randomized porcine microdialysis study
Source: Cancer Chemother Pharmacol. 2024 Feb 8;93(6):555–64. doi: 10.1007/s00280-023-04637-1 (PMC11130026; doi:10.1007/s00280-023-04637-1)

Supplemental figure 1

A

MRM chromatograms of reference standard (left) and microdialysate (right) samples with doxorubicin, doxorubicinol and stable isotope labeled internal standard 13CD3-doxorubicin traces.

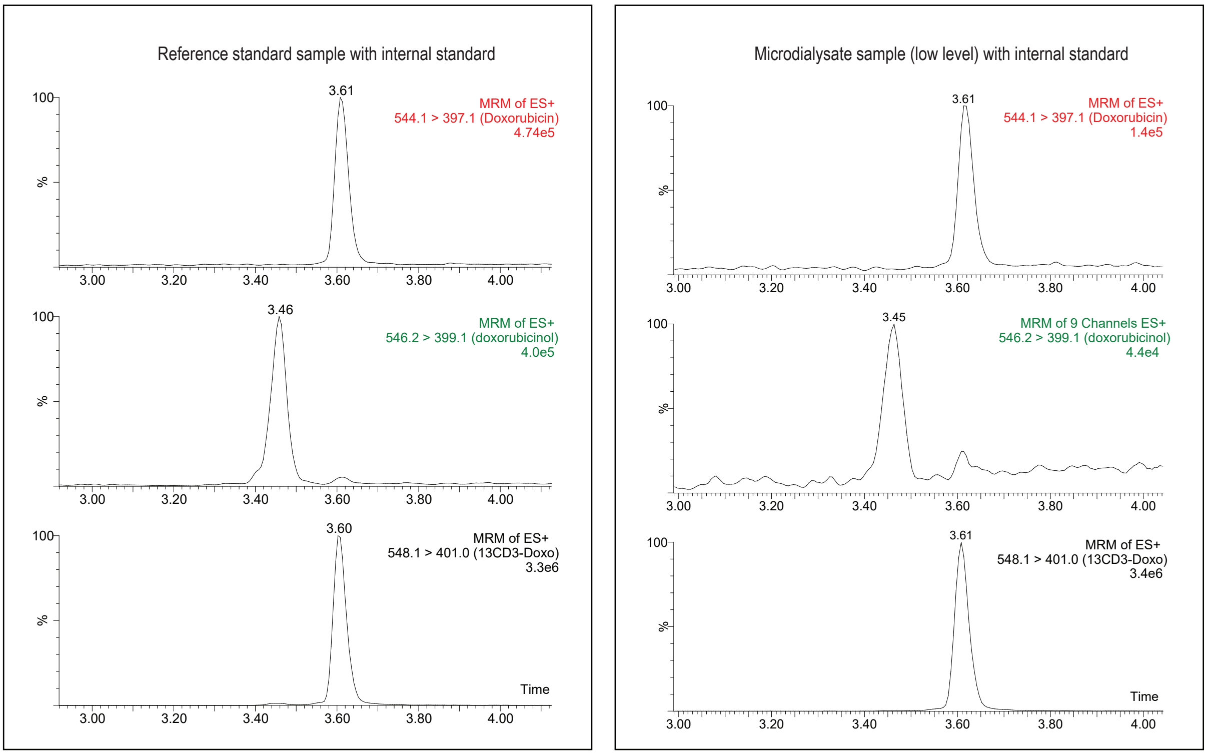

B

Product ion scan (ms/ms) analysis of doxorubicin (left) and doxorubicinol (right) after collision induced dissociation at collision energy 15eV.

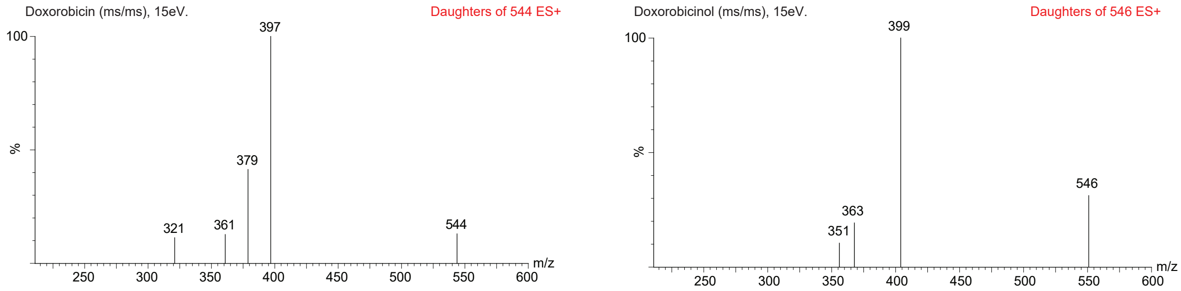

C

Linear calibration model used for doxorubicin quantification. The peak area ratio (area analyt / area internal standard) is plotted against the doxorubicin concentrations of calibrator samples. Linear regression with 1/x weighting performed (graphpad prism) and regression parameters added to plot.

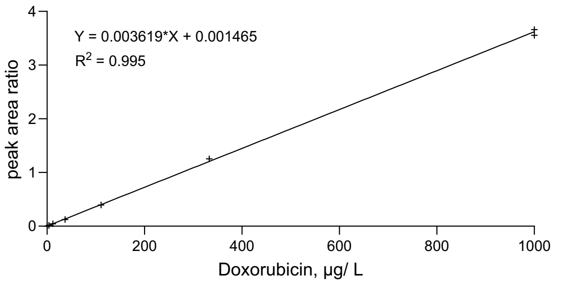

Supplement: Supplementary file 1 — Supplementary file1 (PDF 542 KB) [file 280_2023_4637_MOESM1_ESM.pdf]
